# Supplementary material for: Novel Spiro-Core Dopant-Free Hole Transporting Material for Planar Inverted Perovskite Solar Cells
Source: Nanomaterials (Basel). 2023 Jul 10;13(14):2042. doi: 10.3390/nano13142042 (PMC10385314; doi:10.3390/nano13142042)
Supplement: Supplementary file 1 [file nanomaterials-13-02042-s001.zip › nanomaterials-2481350-supplementary.pdf]

Supporting Information

Novel Spiro-Core Dopant-Free Hole Transporting Material for Planar Inverted Perovskite Solar Cells

Raquel Royo [1], José G. Sánchez [2], Wenhui Li [2], Eugenia Martinez-Ferrero [2], Emilio Palomares [2,3,*], Raquel Andreu [1] and Santiago Franco [1,*]

[1] Instituto de Nanociencia y Materiales de Aragón (INMA), Departamento de Química Orgánica, CSIC-Universidad de Zaragoza, 50009 Zaragoza, Spain; raquelroyo@posta.unizar.es (R.R.); randreu@unizar.es (R.A.)
[2] Institute of Chemical Research of Catalonia, The Barcelona Institute of Science and Technology (ICIQ-BIST), Avinguda Països Catalans 16, 43007 Tarragona, Spain; jgsanchez@iciq.es (J.G.S.); wli@iciq.es (W.L.); emartinez@iciq.es (E.M.-F.)
[3] Catalan Institution for Research and Advanced Studies (ICREA), Passeig Lluïs Companys, 23, 08010 Barcelona, Spain
* Correspondence: epalomares@iciq.es (E.P.); sfranco@unizar.es (S.F.)

Synthesis

All reagents and starting materials were obtained from commercial sources and used as received. Infrared measurements were carried out in KBr using a Nicolet model 360 Avatar FT-IR spectrometer. ^{1}H-NMR spectra were recorded on a Bruker AV300 or a Bruker AV400 at 300MHz or 400 MHz respectively. ^{13}C-NMR spectra were recorded on a Bruker AV300 or a Bruker AV400 at 75 MHz or 100 MHz respectively.

2-((4-methoxyphenyl)amino)ethan-1-ol (1)[1,2]

CuCl (40 mg, 0.4 mmol), 4-iodoanisole (1.2 g, 5.0 mmol) and KOH (448 mg, 8.0 mmol) were added to a screw-capped test tube. Then, 980 µl (16.0 mmol) of ethanolamine was added dropwise at 0 ºC and after 10 min at this temperature, the reaction mixture was stirred overnight at room temperature. The crude was diluted with 2 mL of water before extraction with ethyl acetate (3x10 mL), and dried over $MgSO_4$. After removing the solvent, the crude alcohol was purified by silica gel column chromatography (hexane/ethyl acetate 1:1) to afford the desired product (795 mg, 96 %).

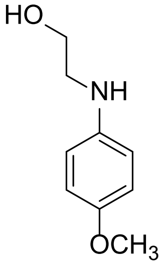

Chemical Formula: $C_9H_{13}NO_2$

Molecular weight: 167.21 g/mol

^{1}H NMR (400 MHz, Chloroform-*d*) δ (ppm): 6.82 – 6.77 (m, 2H), 6.66 – 6.62 (m, 2H), 3.82 (t, *J* = 5.7, 4.7 Hz, 2H), 3.75 (s, 3H), 3.26 (t, 2H).

4-methoxy-N-(2-((triisopropylsilyl)oxy)ethyl)aniline (2)[3]

Triisopropylsilyl chloride (1 mL, 4.7 mmol) and imidazole (870 mg, 12.8 mmol) was added to a solution containing *p*-methoxiphenylaminoethanol (712 mg, 4.3 mmol) in CH_2Cl_2 (10 mL). The reaction mixture was stirred at room temperature in argon atmosphere for 3 hours. Then, the reaction was washed with sat. NaCl (3x10 mL) and the organic layer was dried over $MgSO_4$ and concentrated in vacuum. The crude was purified by silica gel chromatography (hexane:ethyl acetate 9:1) to yield the target compound (1.27 g, 92 %).

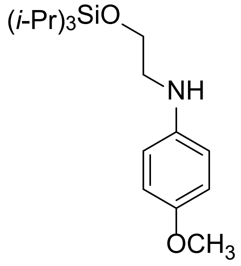

Chemical Formula: $C_{18}H_{33}NO_2Si$

Molecular weight: 323.55 g/mol

IR (KBr) ν (cm^{-1}): 3388 (N-H), 2942 (=C-H), 2865(-C-H), 1619, 1513, 1463 (C=C), 1236 (C-O), 819 (para sustitution). ^{1}H NMR (400 MHz, Chloroform-*d*) δ (ppm): 6.79 (d, *J* = 8.9 Hz, 2H), 6.62 (d, *J* = 8.9 Hz, 2H), 3.90 (t, *J* = 5.3 Hz, 2H), 3.75 (s, 3H), 3.19 (t, *J* = 5.3 Hz, 2H), 1.09 – 1.04 (m, 21H). ^{13}C NMR (75 MHz, $CDCl_3$) δ (ppm): 152.69, 143.08, 115.30, 114.87, 62.34, 56.10, 47.62, 18.15, 17.86, 12.29. HRMS(ESI^+) m/z: 324.2353 $[M+H]^+$; calculated for $C_{18}H_{33}NO_2Si$: 324.2353.

N2,N2',N7,N7'-tetrakis(4-methoxyphenyl)-N2,N2',N7,N7'-tetrakis(2 ((triisopropylsilyl)oxy)ethyl)-9,9'-spirobi[fluorene]-2,2',7,7'-tetraamine (3)[4]

In a 100 mL two-necked flask, 2,2',7,7'-tetrabromo-9,9'-spirobi[fluorene] (526 mg, 0.8 mmol), sodium *tert*-butoxide (500 mg, 5.0 mmol), tris(dibenzylideneacetone)dipalladium(0) (332 mg, 0.035 mmol) and 4-methoxy-N-(2-((triisopropylsilyl)oxy)ethyl)aniline (1.27 g, 3.9 mmol) were dissolved in 20 mL of desoxigenated toluene under argon atmosphere. Next, 56 µl of *tert*-butylphosphine was added into the flask and the reaction mixture was heated to reflux at 110 ºC for 12 h. After cooling to room temperature, the reaction crude was filtered in celite before extraction with ethyl acetate and brine water and dried over $MgSO_4$. After the solvent was

evaporated, the resiude was purified by column chromatography (hexane: ethyl acetate 9.5:0.5) to afford a brown-dark oil (695 mg, 79 %).

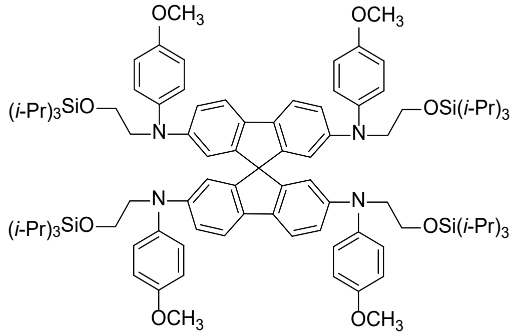

Chemical Formula: $C_{97}H_{140}N_4O_8Si_4$

Molecular weight: 1602.55 g/mol

IR (KBr) ν (cm^{-1}): 3388 (aromatic Csp2-H); 2959, 2851 (aliphatic Csp3-H); 1616, 1595, 1516 (vibrational tension *para* aromatic system, C=C); 1236, 1466 (C-O). ^{1}H NMR (300 MHz, Acetone) δ 7.47 (d, *J* = 8.3 Hz, 1H), 7.06 – 6.98 (m, 2H), 6.81 (d, *J* = 9.0 Hz, 2H), 6.78 – 6.75 (m, 1H), 6.38 (d, *J* = 2.2 Hz, 1H), 3.76 (d, *J* = 9.8 Hz, 7H), 0.97 (d, *J* = 2.1 Hz, 21H). ^{13}C NMR (75 MHz, Acetone) δ 156.49, 151.56, 148.64, 142.04, 134.56, 125.77, 120.16, 118.20, 115.34, 113.78, 61.01, 55.69, 55.61, 18.42, 12.64.

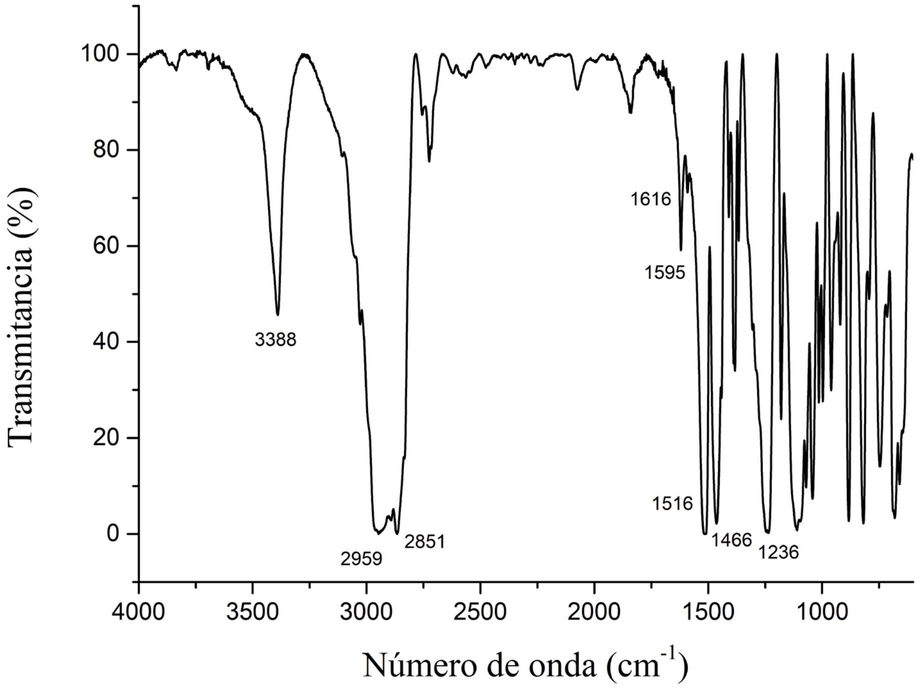

Figure S1: Infrared spectra in KBr of Syl-SC compound.

a)

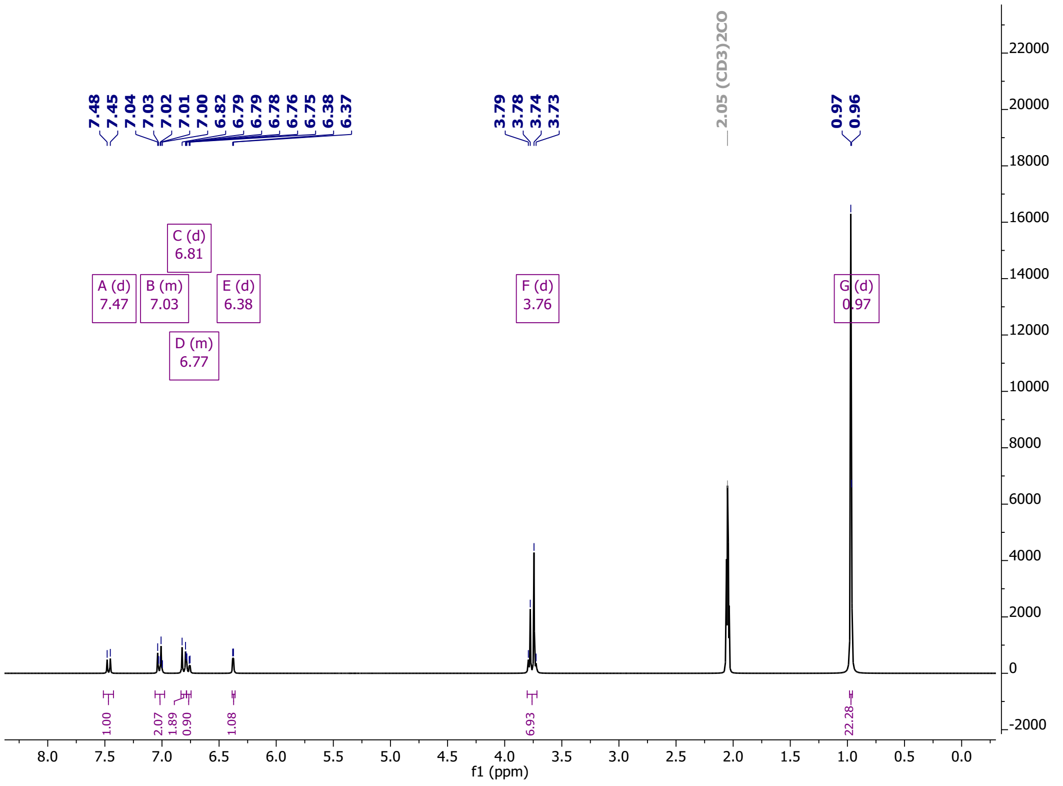

b)

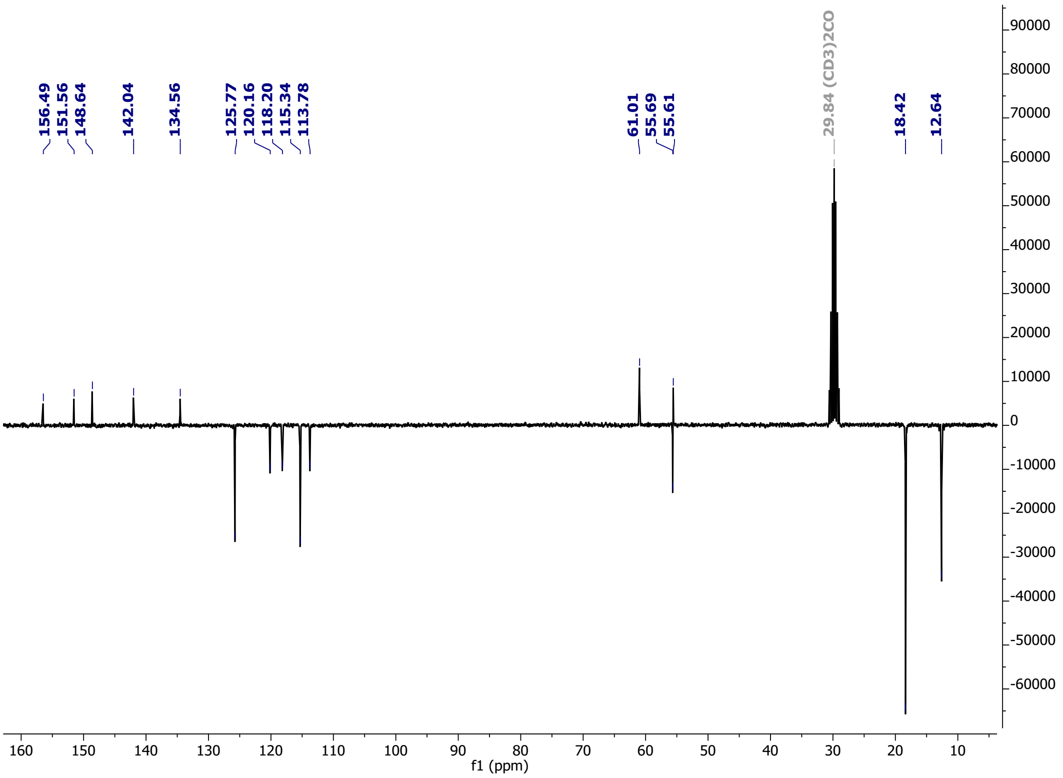

Figure S2: a) ^{1}H NMR spectrum of Syl-SC (300 MHz, CD_3COCD_3), b) ^{13}C NMR spectrum of Syl-SC (75 MHz, Acetone).

Thermal analysis of Syl-SC

Thermogravimetric analysis (TGA) determines the rate and the weight loss of a phase as a function of temperature or time under a controlled atmosphere. The measurements are carried out in a Mettler Toledo TGA/SDTA851 equipment. The working temperature went from 30 ºC to 900 ºC at a scan rate of 10 ºC/min under nitrogen atmosphere. The decomposition temperature (T_{des}) is considered when the samples loss up to 5% of its initial weight.

Differential scanning calorimetry (DSC) analysis provides information about physical and chemical changes that involve endothermic and exothermic processes, or changes in the heat capacity. DSC analysis are measured in a Mettler Toledo DSC822 calorimeter with a 56-point Au-AuPd thermopile FRS5 sensor. The working temperature went from 30 ºC to 300 ºC performing three continuous cycles, at a scan rate of 10 ºC/min. The glass transition temperatires for the investigation compounds are determined during the first heating scan under nitrogen atmosphere.

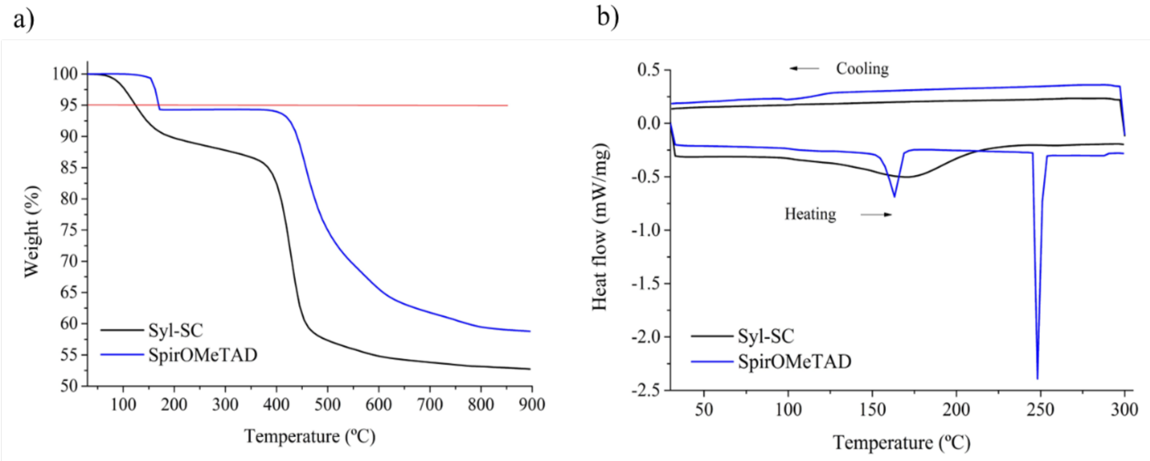

Figure S3: (a) Thermogravimetric analysis (TGA) curve of Syl-SC; method 30-900 ºC to 10ºC/min under 80.0 ml/min of N_2 (b) Differential scanning calorimetry (DSC) trace of Syl-SC measured under N_2 flow at heating and cooling of 10ºC/min from 30 to 300 ºC.

Table S1: Thermal properties of HTM.

**Compound**	**T_{des} (ºC)[a]**	**T_g/(T_f) (ºC)[b]**
Syl-SC	123	171
SpirOMeTAD	170	163/(247)

[a] Decomposition temperature determined from TGA (5 % weight loss). [b] Glass transition (Melting point) temperature determined from the first cycle of DSC.

Opto-electrochemical measurements

UV-Visible spectra were recorded with a Cary 6000i UV-Vis-NIR spectrophotometer. Differential Pulse Voltammetry and cyclic voltammetry (CV) measurements were performed with a μ-Autolab type III potentiostat using a glassy carbon working electrode, Pt counter electrode, and Ag/AgCl reference electrode. The experiments were carried out under argon in compound solutions $5 \cdot 10^{-4}$ M CH_2Cl_2, with Bu_4NPF_6 as supporting electrolyte (0.1 mol L^{-1}). Scan rate was 0.01 V s^{-1}, modulation amplitude 0.025V and modulation time 0.05 s^{-1}.

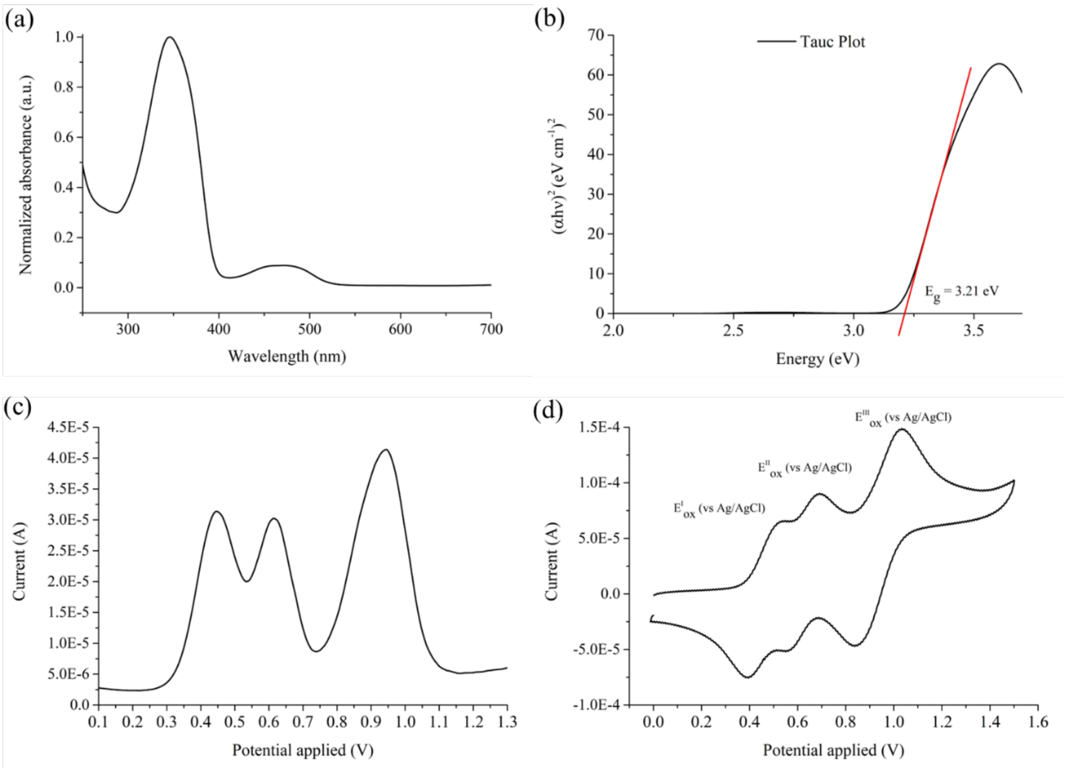

Figure S4: (a) UV-vis of Syl-SC in CH_2Cl_2 solution. (b) Tauc Plot to estimate the band gap (E_g) of the Syl-SC molecule. (c) Differential pulse voltammetry (DPV) and (d) cyclic voltammetry in CH_2Cl_2 solution for Syl-SC vs Ag/AgCl.

Table S2: Optical and electrochemical properties of Syl-SC.

HTM	λ_{abs} (nm)	E_g (eV)[a]	E^{I}_{ox}(V)[b]	E^{II}_{ox}(V)[b]	E^{III}_{ox}(V)[b]	E_{HOMO}(eV)	E_{LUMO}(eV)
Syl-SC	345	3.21	+0.66	+0.89	+1.23	-5.46	-2.32

[a] Band gap estimated from the Tauc plot. [b] The oxidation potentials, measured in DCM solution 0.1 M $TBAPF_6$ as the electrolyte, Ag/AgCl as reference electrode and Pt as counter electrode respectively, were converted to normal electrode (NHE) by addition of 0.199 V.

Photovoltaic measurements

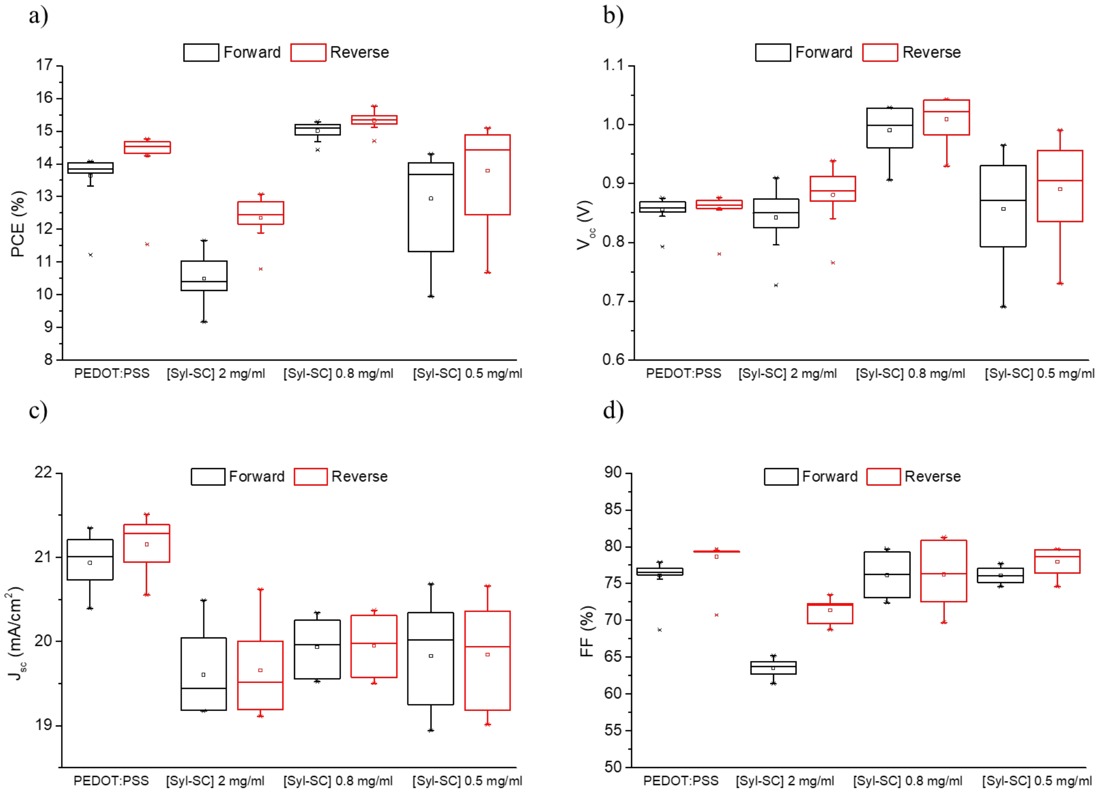

Figure S5: Devices based on PEDOT:PSS and Syl-SC at different concentrations: (a) Performance device (PCE) boxplot; (b) Open circuit voltages (V_{oc}) boxplot; (c) Current density (J_{sc}) boxplot and (d) Fill factor (FF) boxplot.

Contact angle and field emission scanning electron microscopy (FESEM)

Field emission scanning electron microscopy (FESEM) was used with an FEI Quanta 600 microscope to obtain the surface morphology of perovskites. Contact angle measurements were performed with an optical tensiometer (Attention Theta Flex, Biolin Scientific, Sweden) using a sessile drop analysis.

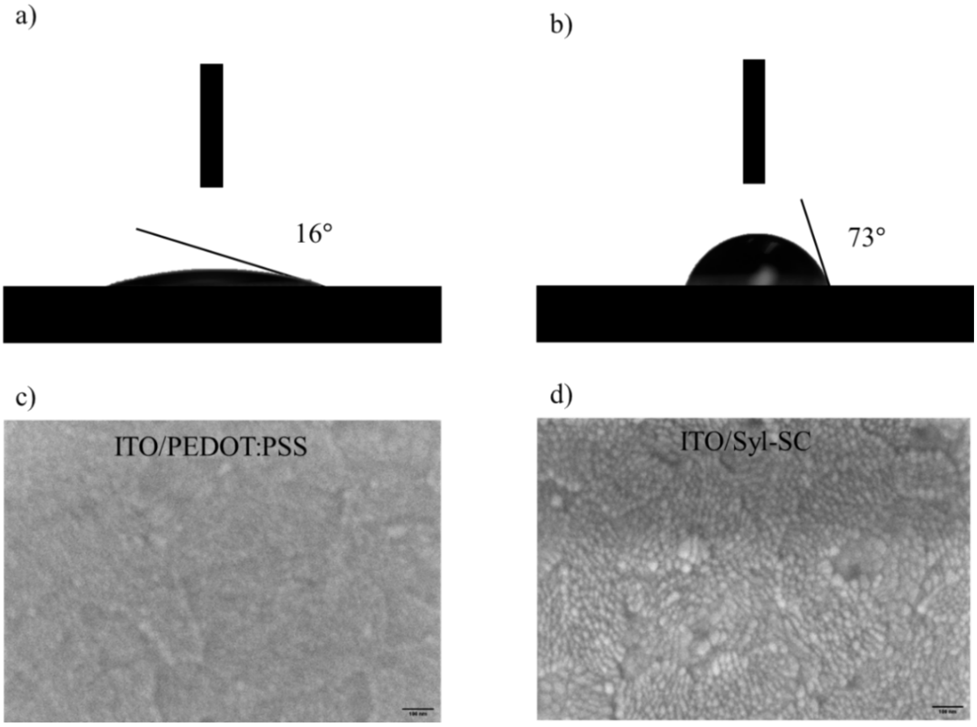

Figure S6: The contact angles between HTL film and water droplet on the substrate of a) PEDOT:PSS and b) Syl-SC. Top surface FESEM of c) PEDOT:PSS and d) Syl-SC.

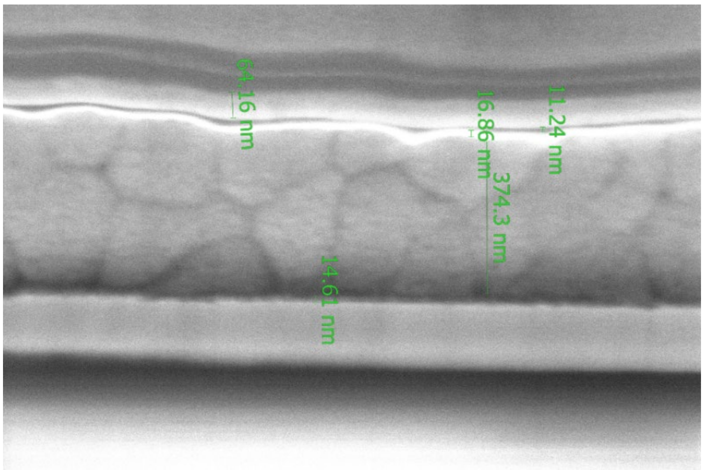

Figure S7: Cross-sectional FESEM image of the p-i-n device containing Syl-SC as HTM with the thickness of the layer's components.

Electrochemical impedance spectroscopy (EIS) measurements

Table S3. Impedance parameters extracted from Nyquist plots fitted with the electrical equivalent circuit.

HTM	R_{series} [Ω]	R1 [Ω]	C1 [nF]
Syl-SC (0.8 mg/ml)	7.30	35.01	4.97
PEDOT:PSS	7.18	25.11	8.06

Transient optoelectronic measurements

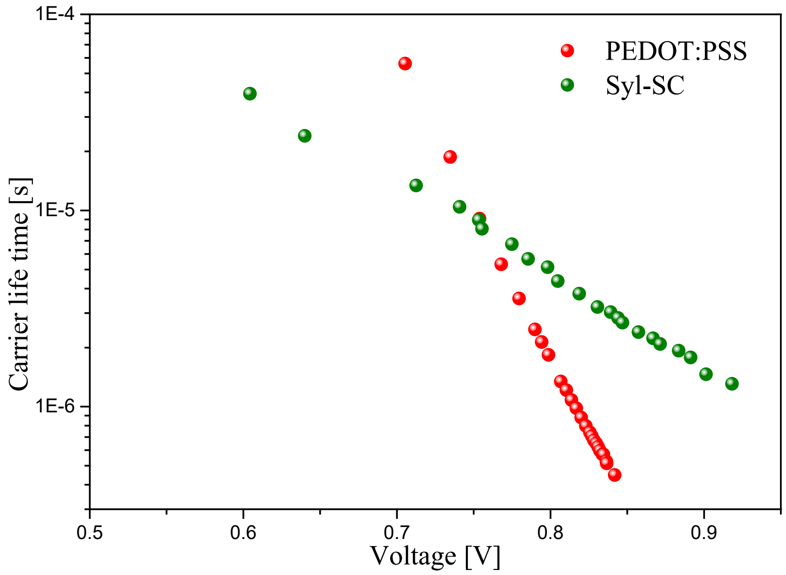

Figure S8: Charge carrier lifetime ($\tau_{\Delta n}$) as a function of device Voc.

References

1. Yin, H.; Jin, M.; Chen, W.; Chen, C.; Zheng, L.; Wei, P.; Han, S. Solvent-Free Copper-Catalyzed N-Arylation of Amino Alcohols and Diamines with Aryl Halides. *Tetrahedron Lett* **2012**, *53*, 1265–1270, doi:10.1016/j.tetlet.2011.12.130.

2. Martina, K.; Rinaldi, L.; Baricco, F.; Boffa, L.; Cravotto, G. Highly Efficient Mechanochemical N-Arylation of Amino Alcohols and Diamines with Cu0 Powder. *Synlett* **2015**, *26*, 2789–2794, doi:10.1055/s-0035-1560173.

3. Novel Molecules That Selectively Inhibit Histone.

4. Jeon, N.J.; Lee, H.G.; Kim, Y.C.; Seo, J.; Noh, J.H.; Lee, J.; Seok, S. il O-Methoxy Substituents in Spiro-OMeTAD for Efficient Inorganic-Organic Hybrid Perovskite Solar Cells. *J Am Chem Soc* **2014**, *136*, 7837–7840, doi:10.1021/ja502824c.
